# Supplementary material for: An EDS1 heterodimer signalling surface enforces timely reprogramming of immunity genes in Arabidopsis
Source: Nat Commun. 2019 Feb 15;10:772. doi: 10.1038/s41467-019-08783-0 (PMC6377607; doi:10.1038/s41467-019-08783-0)
Supplement: Supplementary file 1 — Supplementary Information [file 41467_2019_8783_MOESM1_ESM.pdf]

Supplementary Table 1: List of DEG between R493A and *eds1-2* at 8 hpi with *Pst AvrRps4*

**GeneID**	**Symbol**	**Description**	**log2FC.R493A_eds1-2_8hpi**	**FDR.R493A_eds1-2_8hpi**
AT1G54420		unknown protein	7.01	2.16E-09
AT2G33835	FES1	Protein FRIGIDA-ESSENTIAL 1	-5.82	3.30E-09
AT4G16870		transposable element gene	-5.69	1.78E-08
AT1G54410		dehydrin family protein	-9.56	6.31E-07
AT3G57950		unknown protein	5.91	2.74E-04
AT4G08115		transposable element gene	-4.56	6.57E-04
AT4G16910		transposable element gene	-3.39	1.77E-03
AT5G10140	FLC	MADS-box protein FLOWERING LOCUS C	-6.86	2.05E-03
AT5G13320	PBS3	4-substituted benzoates-glutamate ligase GH3.12	3.70	2.65E-03
AT1G30190		unknown protein	5.30	5.53E-03
AT3G48090	EDS1	Protein EDS1	6.03	9.92E-03
AT4G04410		transposable element gene	-2.41	4.64E-02

Supplementary Table 2: Summary of total DEG between *Pst AvrRps4* and *Pst* ΔCor *AvrRps4* treatments for each genotype at 8 hpi and 24 hpi

Comparison	up (p<0.05)	down (p<0.05)	up (l2fc>1, p<0.05)	down (l2fc<-1, p<0.05)
C08vsA08_*eds1-2*	1781	2061	1551	1259
C24vsA24_*eds1-2*	531	922	459	528
C08vsA08_cEDS1	7	74	7	68
C24vsA24_cEDS1	0	3	0	3
C08vsA08_R493A	811	520	708	301
C24vsA24_R493A	113	160	103	107

C08 - *pst ΔCOR AvrRps4* - 8 hpi
C24- *pst ΔCOR AvrRps4* - 24 hpi
A08 - *pst AvrRps4* - 8 hpi
A24- *pst AvrRps4* - 24 hpi

Supplementary Table 3: List of primers used in this study

Name	Sequence	Purpose
K387A_F	gaggtttttaaaggcactagcatggatag	site-directed mutagenesis
K387A_R	ctatccatgctagtgcctttaaaaacctc	site-directed mutagenesis
K487A_F	gggccgtacatgGCaagaggaagaccaac	site-directed mutagenesis
K487A_R	gttggtcttcctcttGCcatgtacggccc	site-directed mutagenesis
K478A_F	catcgacatttaGCgaacgaagacacagg	site-directed mutagenesis
K478A_R	cctgtgtcttcgttcGCtaaatgtcgatg	site-directed mutagenesis
R488A_F	ggccgtacatgaaaGCaggaagaccaacc	site-directed mutagenesis
R488A_R	ggttggtcttcctGCtttcatgtacggcc	site-directed mutagenesis
R493A_F	ggaagaccaaccGCctacatatatgctcag	site-directed mutagenesis
R493A_R	ctgagcatatatgtagGCggttggtcttcc	site-directed mutagenesis
R493K_F	gaggaagaccaaccAAGtacatatatgctc	site-directed mutagenesis
R493K_R	gagcatatatgtaCTTggttggtcttcctc	site-directed mutagenesis
R493E_F	gaggaagaccaaccGAGtacatatatgctc	site-directed mutagenesis
R493E_R	gagcatatatgtaCTCggttggtcttcctc	site-directed mutagenesis
KK440/1-AA_F	GTGCTCGGTTTATTGgcGgcATGTCAACTTCC	site-directed mutagenesis
KK440/1-AA_R	GGAAGTTGACATgcCgcCAATAAACCGAGCAC	site-directed mutagenesis
R420A_F	GGAATAGACCTAAAgcGTATGAGGTGATTG	site-directed mutagenesis
R420A_R	CAATCACCTCATACgcTTTAGGTCTATTCC	site-directed mutagenesis
PR1_F	TTCTTCCCTCGAAAGCTCAA	qPCR
PR1_R	AAGGCCCACCAGAGTGTATG	qPCR
VSP1-F	tcatactcaagccaaacgg	qPCR
VSP1-R	ATCCTCAACCAAATCAGC	qPCR
JAZ10-F	TTATGAAGGTCGCTAATGAA	qPCR
JAZ10-R	ATGGGAAGATCTCCTTCTAG	qPCR
PAD4-F	TCAGTTAAAGATCAAGGAAGGA	qPCR
PAD4-R	GGCGGAGAAGATTGAGATAGA	qPCR
EDS1-F	AGATTATTCAGGTGATCGAGCA	qPCR
EDS1-R	TTTATGGGCTTGACACTTTGG	qPCR
ICS1-F	TACTAACCAGTCCGAAAGACG	qPCR
ICS1-R	GAGGCTTGACAACAACTCTGT	qPCR
bsmt1-F	AGTCTTTGAGGGTCTTGTGAG	qPCR
bsmt1-R	GTCATCTTCTTCGTAGTAGTG	qPCR

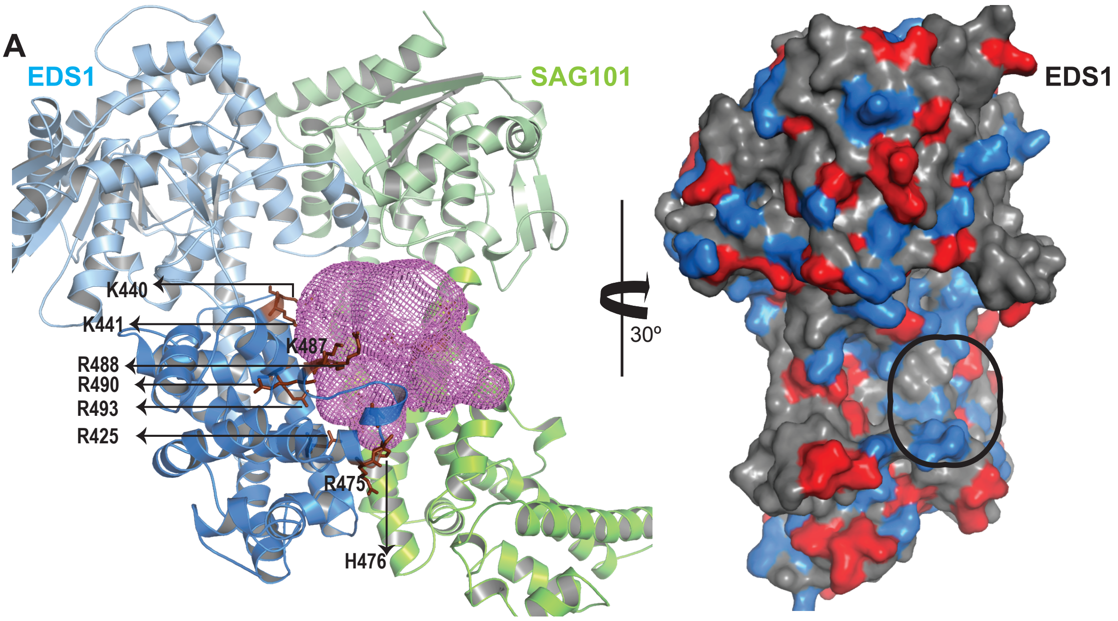

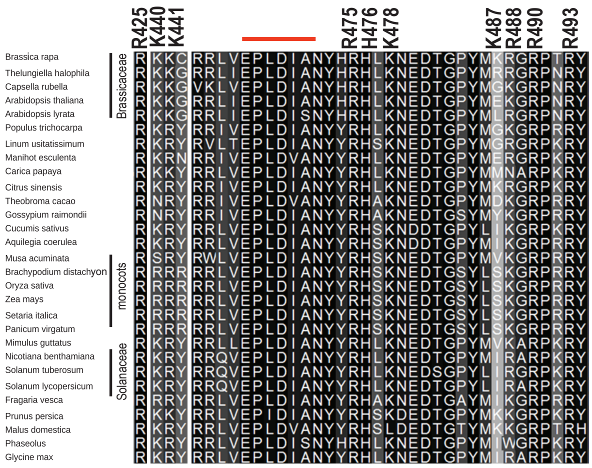

C

RPP4 resistance (*Hpa EMWA1*)	
**Line**	**T1 (resistant/total plants)**
Col-0	20/20
eds1-2	0/24
cEDS1 (WT)	16/16
K387A	24/24
K440A/K441A	4/24
K478A	1/24
K487A	20/20
R488A	24/24
R493A	0/28

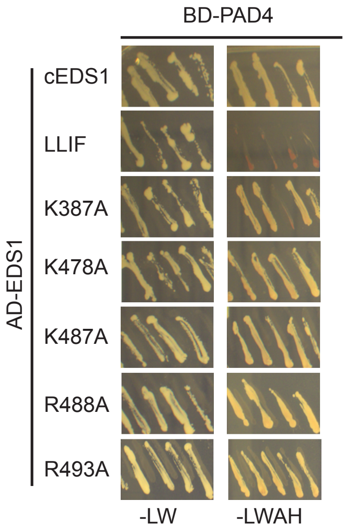

Supplementary Figure 1

A. Crystal structure of EDS1 (blue) - SAG101 (green) heterodimer with cavity formed by the EP-domains (magenta mesh) highlighted. Conserved positively charged residues lining the cavity are depicted as brown sticks. The right panel depicts an electrostatic representation and positive nature of the cavity highlighted within a black oval. **B.** Conservation of EDS1 EP-domain residues across EDS1 orthologs. Sequences were aligned using MUSCLE and conserved amino acid residues in the EP-domain cavity are highlighted, including the characteristic "EPLDIA" conserved motif of the EP-domain. **C.** Summary of TNL (*RPP4*) complementation assay in T1 plants expressing EDS1-YFP EP-domain mutants. For each EDS1 mutant line, individual BASTA-resistant T1 seedlings were monitored for TNL-triggered resistance to *Hpa* EMWA1 (at 5 dpi). Seedlings showing conidospores on leaves were scored as disease susceptible. Numbers of resistant / total plants tested is shown. Non-transgenic controls for *RPP4* resistance Col-0 and *eds1-2* were not treated with BASTA. **D.** Y2H interactions between activation domain (AD) fusions of EDS1 variants and PAD4 (BD) binding domain fusions. The EDS1-LLIF mutant which does not bind PAD4 was used as a negative control. Yeast viability (-LW) and protein interaction (-LWAH) are shown in the GAL4 matchmaker Y2H system.

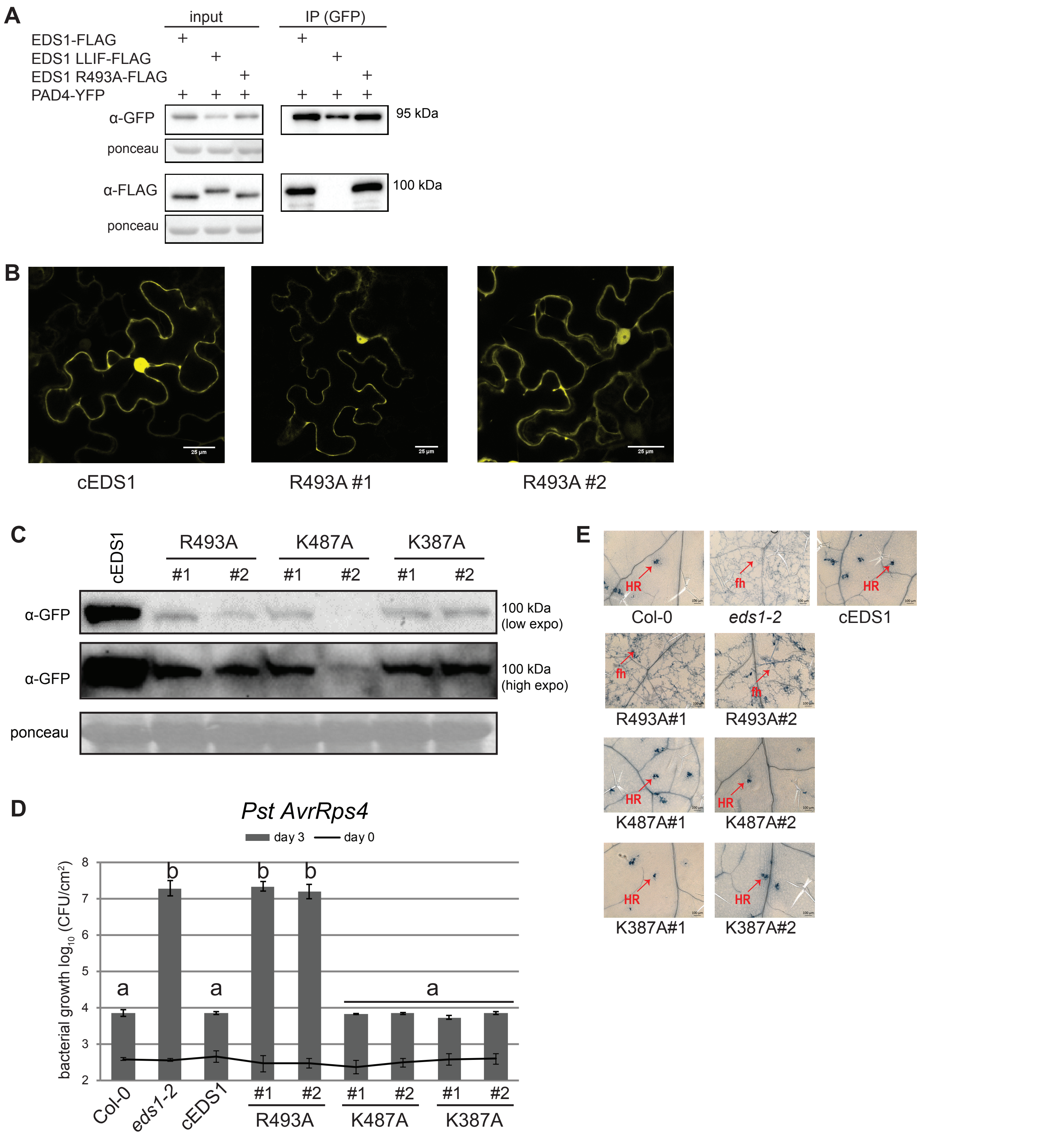

Supplementary Figure 2

A. cEDS1-FLAG variants transiently co-expressed with cPAD4-YFP in *eds1-2 pad4-1 sag101-3* protoplasts (input) were immuno-precipitated (IP) using α-GFP beads. Co-immunoprecipitated FLAG-tagged cEDS1 variants were detected using α-Flag antibodies. EDS1-LLIF which (migrates higher) and does not bind PAD4 was used as a negative control. **B.** Confocal images of four-week-old leaves of transgenic *eds1-2* lines expressing YFP-tagged EDS1 or R493A, showing nucleocytoplasmic localization. Images were taken at 24 hpi with *Pst AvrRps4.* Images are representative of >30 cells per line. Scale bar = 25µm. **C.** Accumulation of EDS1-YFP protein in mock and *Pst AvrRps4* (24 hpi) treated plants of cEDS1, gEDS1 and respective R493A mutants on immunoblots probed using α-GFP antibody. **D.** Four-week-old *Arabidopsis* plants of the indicated genotypes were infiltrated with *Pst AvrRps4* (OD600 - 0.0005) and bacterial titres determined at 0 and 3dpi. No significant difference was observed at 0 dpi. Bars represent mean of three biological replicates ± SE. Differences between genotypes were analysed using ANOVA (Tukey's HSD, $p<0.005$). Similar results were obtained in three independent experiments. **E.** *RPP4* resistance phenotypes of 2-week-old control and *Arabidopsis* transgenic lines expressing cEDS1 and cEDS1 EP-domain mutants, as indicated. Hpa EMWA1 infected leaves were stained with trypan blue at 4 dpi. Each image is representative of >12 leaves from two independent experiments. HR, hypersensitive response; fh, pathogen free hyphae.

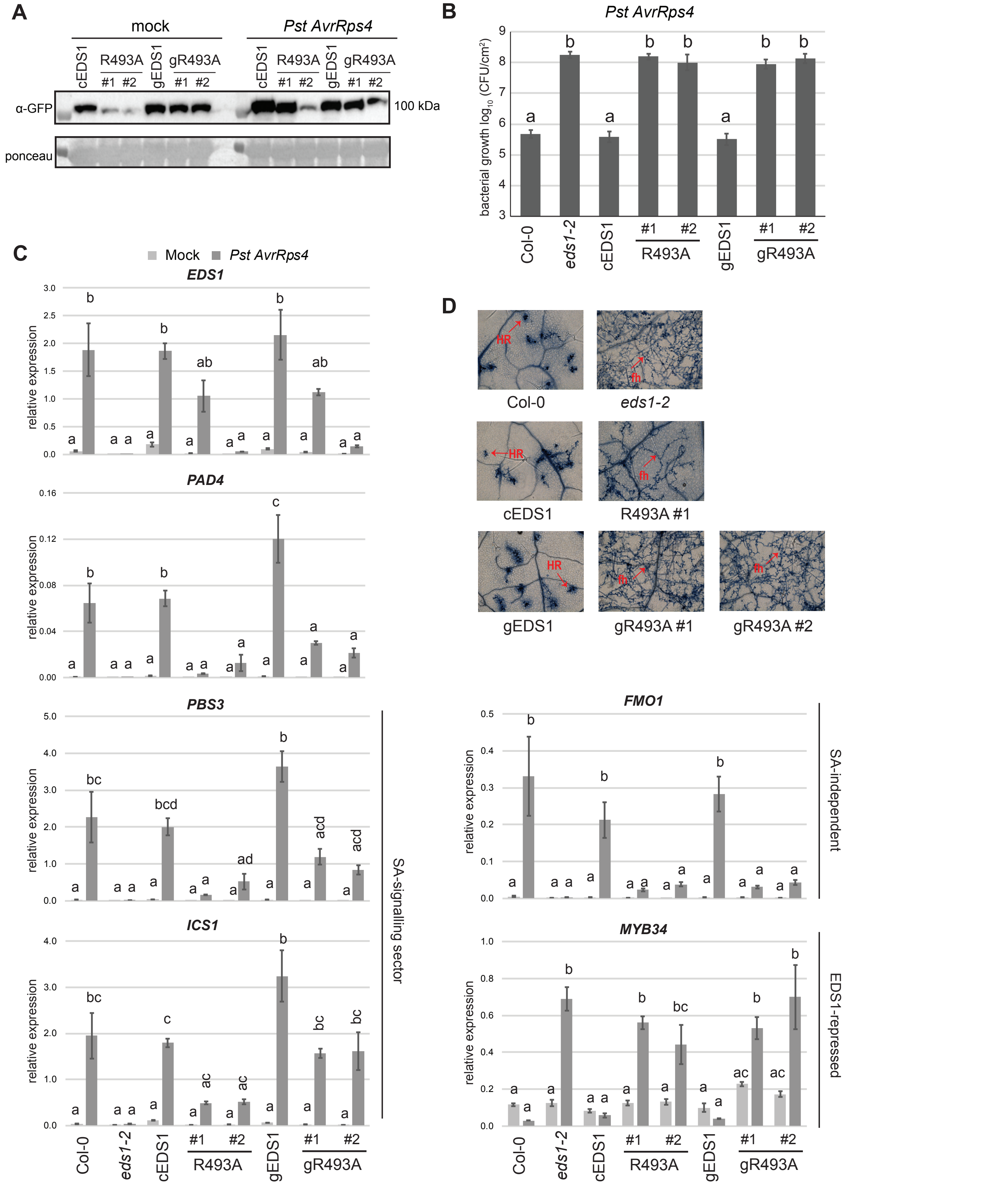

Supplementary Figure 3

A. Accumulation of EDS1-YFP protein in 10mM $MgCl_2$ (mock) and *Pst AvrRps4* (24 hpi) treated plants of cEDS1, gEDS1 and respective R493A mutants on immunoblots probed using α-GFP antibody. **B.** Four-week-old *Arabidopsis* plants of the indicated genotypes were infiltrated with *Pst AvrRps4* (OD600 - 0.0005) and bacterial titres determined at 0 and 3dpi. No significant differences were observed at 0 dpi. Bars represent mean of three biological replicates ± SE. Differences between genotypes were analysed using ANOVA (Tukey's HSD, $p<0.005$). Similar results were obtained in three independent experiments. **C.** Expression of EDS1-dependent marker genes representing different signalling sectors - SA (*PBS3, ICS1*), SA-independent (*FMO1*) and EDS1-repressed (*MYB34*), at 8 hpi with *Pst AvrRps4*, measured by qRT-PCR. Values were normalized to the house-keeping gene *GapDH*. Bars represent means ± SE calculated from two independent experiments each with three biological replicates (n=6). Differences between genotypes were calculated using ANOVA (Tukey's HSD, $p<0.05$). **D.** *RPP4* resistance phenotypes of 2-week-old control and *Arabidopsis* transgenic lines expressing cEDS1, gEDS1 and respective R493A mutants. *Hpa* EMWA1 infected leaves were stained with trypan blue at 5 dpi. Each image is representative of >12 leaves from two independent experiments. HR, hypersensitive response; fh, pathogen free hyphae.

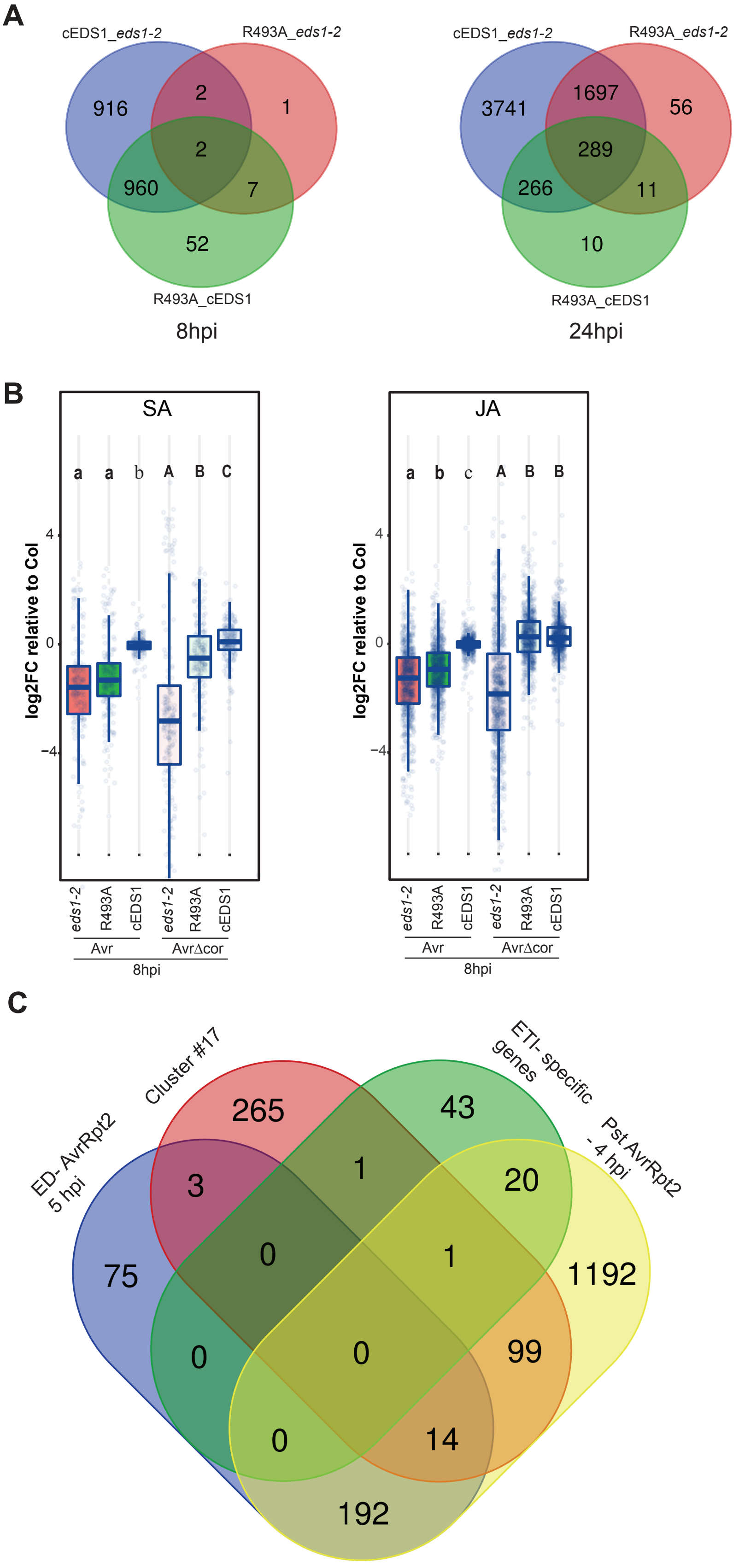

Supplementary Figure 4

A. Venn diagrams presenting overlap of DEGs between the indicated genotypes at 8 and 24 hpi with *Pst AvrRps4*. **B.** Comparison of expression profiles of genes regulated by ICS1/SA[18] and repressed by JA (selected clusters from Hickman et al., 2017 (#15, 18, 19, 20, 23, 24)[48] were chosen based on differential expression profile between cEDS1 and *eds1-2*) with our study using *Pst AvrRps4* and *Pst* Δ *Cor AvrRps4* (ΔCor). Bar plots are coloured based on genotype with a light tone for the *Avr*Δ*Cor* data. Statistical differences between genotypes were analysed within treatments using the Kruskal-Nemenyi test ($p<0.001$) as indicated by different annotations. **C.** A Venn diagram showing overlap between genes in cluster #17 for *Pst AvrRps4* ETI at 8hpi, genes specifically regulated in AvrRpt2-triggered ETI (ETI-specific genes)[9], DEG at 4 hpi with *Pst AvrRpt2* (Pst AvrRpt2 – 4 hpi)[9] and DEG at 5 h with estradiol-induced AvrRpt2 (ED-AvrRpt2-5hpi)[49].

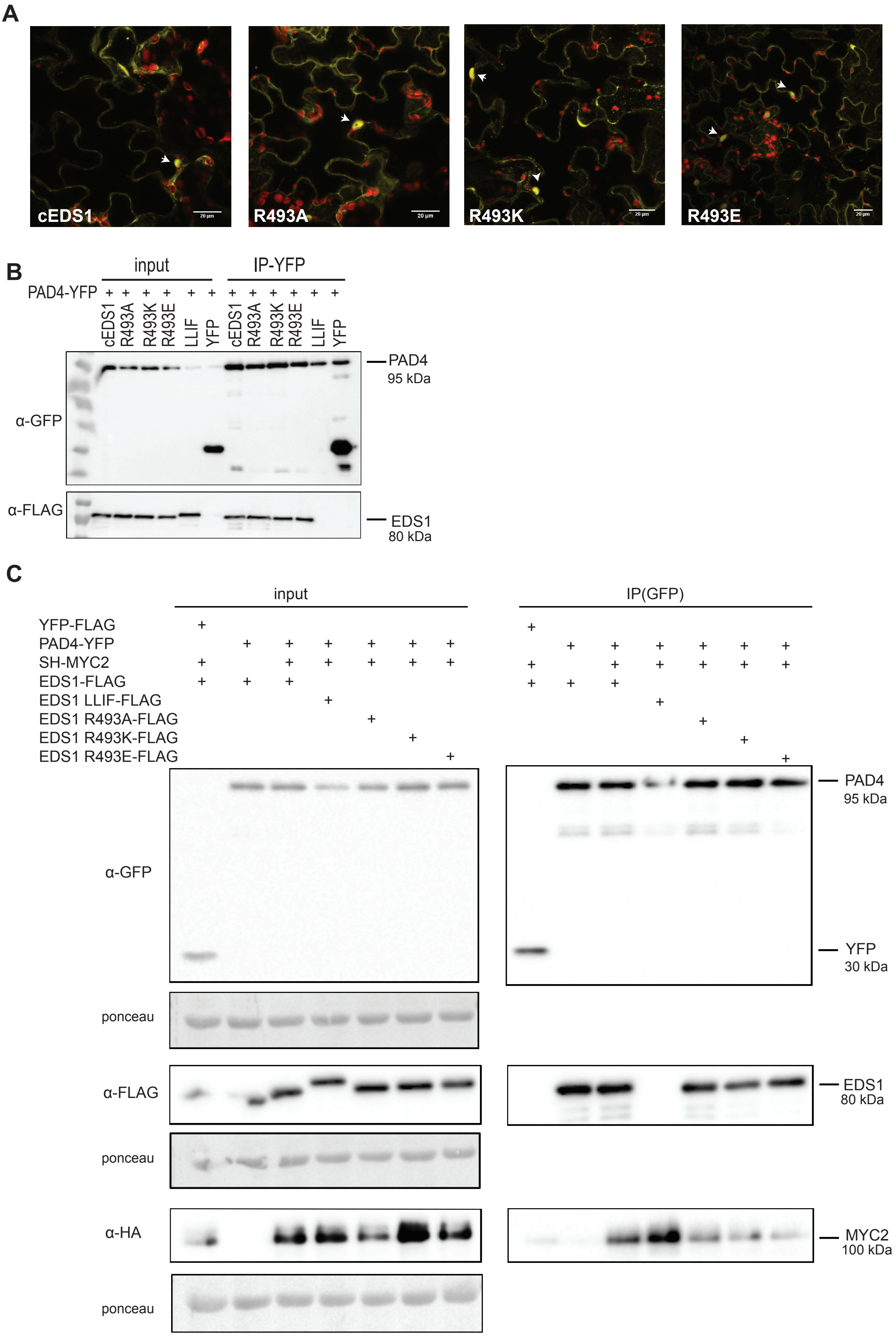

Supplementary Figure 5

A. Confocal images of transiently expressed FLAG-tagged cEDS1 and EDS1^{R493} mutant variants in *N. benthamiana* showing nucleocytoplasmic localization. White arrowheads depict nuclei and chloroplasts fluoresce red. Images are representative of >20 cells/variant at 3d after agroinfiltration. Scale bar = 20µm. **B.** EDS1-FLAG variants transiently expressed with PAD4-YFP in *eds1-2 pad4-1* protoplasts were co-immunoprecipitated with PAD4 using α-GFP beads. Co-IPed EDS1 variants were detected using α-FLAG antibodies. EDS1^{LLIF}-FLAG and YFP-FLAG were used as negative controls in the IP. **C.** EDS1-FLAG variants (as used in B.) were transiently expressed with PAD4-YFP and strep-HA-MYC2 (SH-MYC2) in *eds1-2 pad4-1 sag101-3* protoplasts. PAD4-YFP was IPed using α-GFP beads. Co-IPed FLAG-tagged EDS1 variants and SH-MYC2 were detected on immunoblots using α-FLAG and α-HA antibodies, respectively. EDS1^{LLIF}-FLAG and YFP-FLAG were used as negative controls. IPs were repeated three times with similar results.

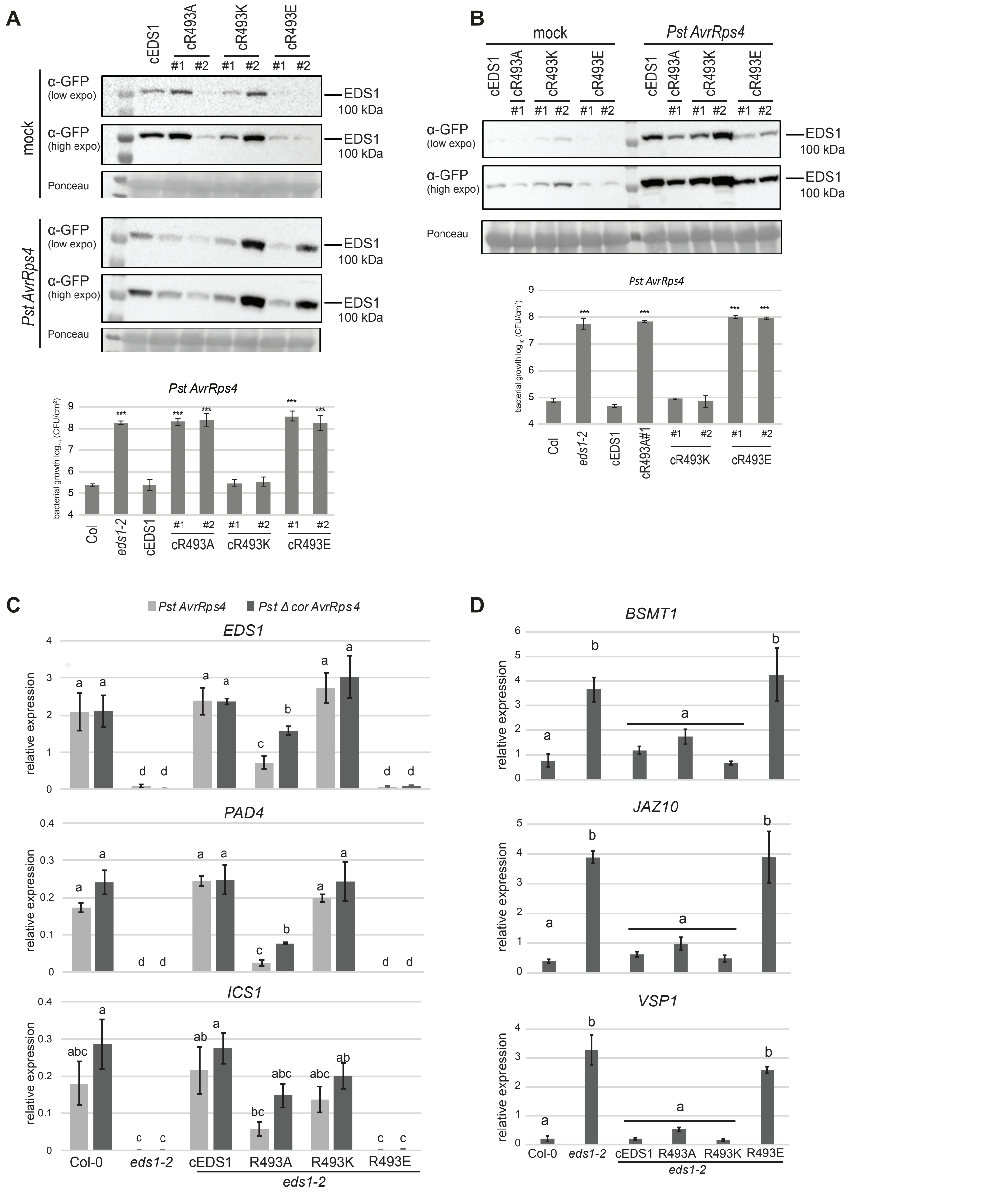

Supplementary Figure 6

A. and B. Accumulation of EDS1-YFP protein in 4-week-old mock (10mM $MgCl_2$) and *Pst AvrRps4* (24 hpi) treated plants of cEDS1 and R493 variants, as indicated, on immunoblots probed using α-GFP antibody. Panels A and B are from independent experiments. Corresponding *Pst AvrRps4* bacterial growth assays (n=2) for experiments A and B are shown. Differences between genotypes were analysed using t-test ($p<0.001$). **C.** Expression of *EDS1*, *PAD4* and *ICS1* in different EDS1 transgenic lines, as indicated, at 8 hpi with *Pst AvrRps4* or *Pst* Δ*cor AvrRps4*, measured by qRT-PCR. Values were normalized to the house-keeping gene *GapDH*. Bars represent means ± SE calculated from three independent experiments. Differences between genotypes were calculated using ANOVA (Tukey's HSD, $p<0.05$). **D.** Expression of selected *MYC2* marker genes *BSMT1*, *JAZ10* and *VSP1* in EDS1 mutant lines at 24 hpi with *Pst AvrRps4,* measured by qRT-PCR. Values were normalized to the house-keeping gene *GapDH*. Bars represent means ± SE calculated from three independent experiments. Differences between genotypes was calculated using ANOVA (Tukey's HSD, $p<0.05$).
