# Supplementary material for: An EDS1 heterodimer signalling surface enforces timely reprogramming of immunity genes in Arabidopsis
Source: Nat Commun. 2019 Feb 15;10:772. doi: 10.1038/s41467-019-08783-0 (PMC6377607; doi:10.1038/s41467-019-08783-0)
Supplement: Supplementary file 3 — Description of Additional Supplementary Files [file 41467_2019_8783_MOESM3_ESM.docx]

Description of Additional Supplementary Files

**Supplementary data 1.** List of genes in cluster #17 (associated with Fig. 4C, D).

**Supplementary data 2.** Gene Ontology (GO) enrichment of cluster #17 genes. GO enrichment was obtained using the panther database.

**Supplementary data 3.** Overlapping genes between cluster #17, BTH-regulated genes and JAregulated genes (associated with Fig. 4D).
